# Adherence to Lifelines Diet Score (LLDS) Is Associated with a Reduced Risk of Breast Cancer (BrCa): A Case-Control Study

**DOI:** 10.1155/2022/7726126

**Published:** 2022-02-04

**Authors:** Mohammad Hassan Sohouli, Mohammad Hadizadeh, Morteza Omrani, Mansoureh Baniasadi, Vahid Sanati, Mitra Zarrati

**Affiliations:** ^1^Cancer Research Center, Shahid Beheshti University of Medical Sciences, Tehran, Iran; ^2^Department of Clinical Nutrition and Dietetics, Faculty of Nutrition and Food Technology, Shahid Beheshti University of Medical Sciences, Tehran, Iran; ^3^Department of Nutrition, School of Public Health, Iran University of Medical Sciences, Tehran, Iran

## Abstract

**Background:**

Previous evidence suggests a link between diet quality and breast cancer (BrCa); however, the link between the Lifelines Diet Score (LLDS)—a fully food-based score that uses the 2015 Dutch Dietary Guidelines—and risk of BrCa has not yet been evaluated. Therefore, the aim of this study was to observe the relationship between adherence to an LLDS and risk of BrCa in Iranian adults.

**Methods:**

In the hospital-based case-control study, 253 patients with BrCa and 267 non-BrCa controls were enrolled. Individual's food consumption was recorded to calculate LLDS using a semiquantitative food frequency questionnaire. In adjusted models, the association between the inflammatory potential of the diet and the risk of BrCa was estimated by using binary logistic regression.

**Results:**

Compared with control individuals, BrCa patients significantly had higher waist circumference (WC), first pregnancy age, abortion history, and number of children. In addition, the mean intake of vitamin *D* supplements and anti-inflammatory drugs in the case group was significantly lower than the control group. Furthermore, after adjusted potential confounders, individuals in the highest vs. lowest quartiles of LLDS showed statistically significant lower risk of BrCa in overall population (OR: 0.21; 95% CI: 0.11–0.43; P trend <0.001), premenopausal (OR: 0.26; 95% CI: 0.10–0.68; P trend = 0.003), and post-menopausal women (OR: 0.20; 95% CI: 0.06–0.60; P trend = 0.015).

**Conclusion:**

Findings of this study reflected that higher LLDS decreased risk of BrCa, but need further investigation in later studies.

## 1. Introduction

Breast cancer (BrCa) is one of the most common cancers in women that rapidly increasing worldwide [1]. One in eight women in Western societies is reported to suffer from this disease, and this amount is about 22.6 per 100,000 females, among Iranian population [2, 3]. There are two types BrCa risk factors which include intrinsic factors like genetic variations that plays an important role in BrCa incidence and extrinsic factors causing cancer, like lifestyle-related factors such as low physical activity and carcinogenic dietary habits. Meanwhile, the role of unhealthy food patterns such as high-saturated fat and high-sugar diets, and consumption of more high-fat dairy products in breast cancer has been more prominent [4, 5]. Past studies have also found that modifying the individual diet with more intake of fruit and vegetables and reducing the consumption of sweets are one of the best ways to prevent breast cancer [5–10]. But due to the comprehensiveness of dietary patterns and their more reliable association with various chronic diseases, several nutritional indices have emerged. These indices include the Mediterranean Diet Scores (MED) score and the Healthy Eating Index (HEI). One of the main components of these two scores is to consider more fruits and vegetables in the diet, and relationship between these indices and reduced the risk of various diseases such as cancer has been shown [11–15]. Although both scores consider consuming saturated and unsaturated fatty acids, but in the Mediterranean diet, it is recommended to reduce dairy consumption, while previous studies have shown that dairy consumption such as yogurt is inversely related to some cancer risk such as colon and breast cancer [16–18]. Furthermore, in the Mediterranean score, there is not a recommendation about sweetened drinks with diet sugar, the carcinogenic effects of which have been proven in breast cancer [19]. Due to deficiencies such as the fact that explain these two scores are not totally diet-based, a new score called Lifelines Diet Score (LLDS) has emerged. Lifelines Diet Score (LLDS) is a brand new score based on food, which is according to the Dutch Dietary Guidelines, in order to find the association of diseases with diets [17]. In this score, food groups were classified according to LLDS guidelines as foods with positive (vegetables, fruits, whole grain products, legumes and nuts, fish, oil and soft margarine, unsweetened dairy products, coffee, and tea), negative (red and processed meat, butter and hard margarine, and sugar-sweetened beverages), neutral, or unknown effects on health, in which neutral and unknown food groups are not involved in the LLDS calculation [17]. According to our information, so far, no study has been conducted to investigate the effect of LLDS with risk of cancer, especially breast cancer; however, two studies examine the relationship between this score and sleep quality in obese individuals and another study conducted in order to find the relationship between LLDS and type 2 diabetes incidence in the Dutch Lifelines cohort [20, 21]. The results of the two studies showed that higher scores improve sleep quality in obese individuals and reduce the risk of type 2 diabetes in the Dutch Lifelines cohort, respectively [20, 21]. We hypothesized that people who consumed a diet high in fruits and vegetables, as well as eating less red meat and sugary drinks, had a higher LLDS and reduced levels of cancer-causing agents in the body; as a result, the risk of BrCa can be reduced. Thus, the aim of the present study was to investigate the association between LLDS and risk of BrCa in a case-control study.

## 2. Methods

The type of study is a case-control study. The target population of this study was 520 samples (two hundred and fifty-three people diagnosed with BrCa and two hundred and sixty-seven people without the disease). It should be noted that this number of samples were selected from “Hazrat Rasoul and Taleghani Hospital, Tehran, Iran,” in a period of one year between the years 2019 and 2020. The minimum required sample size was calculated based on the ability to detect an OR of 2 with a case to control ratio of 1 : 1, 90% power, and a type I error (*α*) rate of 5% [22].

BrCa patients were newly (<6 months) diagnosed with histologically confirmed BrCa by an oncologist. Patients with a history of other cancers, hormone-related diseases such as PCOS (polycystic ovary syndrome) or endometriosis, and occurrence of metastasis were also excluded from our study. The control group was selected from other parts of the hospital such as eye; ear, nose, and throat; skin; and beauty; did not have any history of cancer (benign and malignant), inflammatory disease, and hormone-related diseases; and for the past 6 months, they have had a regular diet. Matching between two groups was done based on age and BMI. The control group was selected from other departments of the hospital, such as ophthalmology, otolaryngology, dermatology, and aesthetics. The group did not have any history of cancer (benign and malignant), inflammatory disease, and hormone-related diseases. Also, each member of the control group included in the study was examined for inflammation by an internal medicine specialist and laboratory results to ensure the absence of inflammation. In order to determine the level of physical activity of individuals, a valid short form of the International Physical Activity Questionnaire (short IPAQ) was used, and informed written consent was obtained from all patients. Finally, it is important to note that this study was accepted via the Research Council and the Ethics Committee of Iran University of Medical Sciences, Tehran, Iran.

### 2.1. Dietary Assessment

A valid questionnaire of semiquantitative food frequency (including 168 food items) was used to determine food consumption compared to last year [23]. The main feature of this FFQ was to follow the usual Iranian food with standard serving size, and for each food, the regular amount of portion eaten and the number of times consumed by the participant was obtained by filling in the FFQ. Consumption for each food consisted as follows: at no time, two-three times/month, one time/week, two-four times/week, five-six times/week, and every day. Portion sizes were transformed to gram, by typical Iranian family measures [24]. Day-to-day nutrient consumptions for each individual were estimated by use of the United States Department of Agriculture's (USDA) national nutrient databank [25]. Nutritionist IV software was applied to determine the everyday energy and nutrients consumption for each subject.

### 2.2. Assessment of Nondietary Exposures

A skilled questioner managed all other questionnaires. So, all the attendee answered properly to the study queries. Overall appearances and medical data gathered through FFQ, consisted of age (years), age at 1st pregnancy (years), menopausal status (premenopause or postmenopause), literacy status, oral contraceptive medicines intake history (month), history of benign breast illness (yes or no), family history of cancer (yes or no), BrCa household history (yes or no), bra wearing at night (yes or no), smoking (yes or no), supplement consumption (yes or no; if yes, the corresponding info about dosage and intake times), NSAIDs usage (yes or no), and contact to sunlight throughout the day ((1) less than half an hour a day, (2) between half an hour and one hour a day, (3) between one and two hours a day, (4) more than two hours a day). Anthropometric quantities were gotten by means of standardized procedures. Weight was measured while patients wearing comfortable clothes, barefooted by a digital Seca scale (made in Germany) with the precision of one hundred grams. Height was obtained in a standing position barefooted, by means of a tape meter. Body mass index (BMI) was considered as weight (kg) divided by the square of height (m2). Waist circumference (WC) was obtained by means of nonelastic tap at the slimmest level deprived of any heaviness to body surface.

### 2.3. Calculation of Lifelines Diet Score

LLDS was computed originated from the process described in the study by Vinke et al. [17]. In this score, food groups were classified according to LLDS guidelines as foods with positive, negative, neutral, or unknown effects on health, which neutral (eggs) and unknown food (included potatoes, refined grain products, white unprocessed meat, cheese, ready-to-eat savory products, sugary products, soups, sweetened dairy, and artificially sweetened products) groups are not involved in the LLDS calculation. Nine foods including vegetables, fruits, whole grain products, legumes and nuts, fish, oil and soft margarine, unsweetened dairy products, coffee, and tea were considered as foods with positive effects. In addition, foods with negative effects include 3 food groups: red and processed meat, butter and hard margarine, and sugar-sweetened beverages. In order for LLDS to show the quality of the relative diet, taking into account the difference in energy consumption between individuals, food intake was calculated in terms of grams per 1000 kcal (kcal) instead of grams per day for each food group and the individual's results divided into quintiles from one to five, with “one” scores the minimum consumption in each food group and “five” scores the maximum. Summing the scores of the twelve components leads to an LLDS score of twelve to sixty.

### 2.4. Statistical Analyses

Statistical analysis was performed using SPSS 20 software (version 19.0; SPSS Inc., Chicago IL). The normalcy of variables was considered by Shapiro–Wilk exams. Mean values of more than 2 groups were evaluated by means of a nalysis of variance (ANOVA) for normal distribution variables. Furthermore, the chi-square test was intended for comparing categorical variables. The risk of breast cancer (OR) and respective 95% confidence interval (95%CI) were estimated by binary logistic regression analysis with adjustment for confounders. In order to calculate ORs and 95% CIs, we classified all subjects based on the LLDS score into quartile ranges using the rank cases and then entered into the logistic regression as a categorical variable. The overall Ptrend across increasing quartiles was examined by considering the median score in each category as a continuous variable. We adjusted the results in three models using a priori selected potential confounders, which included model 1—age and BMI, model 2—additional adjustment for waist circumference, energy, first pregnancy age, number of children, history of abortion, hormone replacement therapy, use of anti-inflammatory drugs, and vitamin D supplementation. The data were presented as mean ± standard deviation, and statistical significance was accepted, a priori, at *P* < 0.05.

### 2.5. Result

The mean ± SD for the age and BMI of the study population was 47.92 ± 10.33 years and 29.43 ± 5.51 kg.m2, respectively.

Demographic characteristics, lifestyle, and medical history of study participants among case and control groups as well as across quartiles of LLDS are shown in Table 1. Compared with control individuals, BrCa patients significantly had higher waist circumference (WC), first pregnancy age, abortion history, and number of children. In addition, the mean intake of vitamin D supplements, hormone replacement therapy, and anti-inflammatory drugs in the case group was significantly lower than the control group. However, no significant differences were found for other characteristics and variables among cases and controls subjects as well as across quartiles of LLDS.

The mean dietary intake of the participants in the study based on case and control groups as well as quartiles of LLDS is shown in Table 2. Although subjects with BrCa had higher intake of energy, fat, saturated fatty acids (SFA), cholesterol, carbohydrates, sodium, folate, and iron than controls, but they had lower intake of mono- and polyunsaturated fatty acids (MUFA and PUFA), potassium, phosphorus, calcium, vitamin B12, and micronutrients antioxidants such as zinc, magnesium, and vitamins E, C, and D. Also, the intake of protein, potassium, phosphorus, calcium, magnesium, zinc, vitamins C, and D in the highest quartiles of LLDS vs. the lowest quartiles of LLDS significantly increased; however, the intake of fats, MUFA, and PUFA decreased.

Table 3 shows the dietary intake of 12 components LLDS (in grams per 1000 kcal) among study subjects based on the case and control groups as well as quartiles this score. Among the positive components LLDS, BrCa patients significantly had lower consumption of healthy food groups including vegetables, fruits, and legumes and nuts and higher intake of coffee. Furthermore, among the negative components of this score, red and processed meat and sugar-sweetened beverages intake were significantly higher in the case group than the control group. Across quartiles of LLDS, all the positive components of this score except coffee and tea significantly increased and its negative components significantly decreased.

The Odds ratios (ORs) and 95% confidence intervals (CIs) for BrCa patients according to quartiles of LLDS gives in Table 4. In crude and adjusted model 1(age and BMI adjusted), we observed a significant decreased odds ratio of BrCa across quartiles of LLDS in the overall population of women (OR: 0.31; 95% CI: 0.17–0.56; P trend <0.001 and OR: 0.28, 95% CI: 0.15–0.52; P trend < 0.001), premenopausal (OR: 0.35; 95% CI: 0.16–0.77; P trend = 0.003 and OR: 0.38, 95% CI: 0.10–0.68; P trend = 0.003), and post-menopausal women(OR: 0.23; 95% CI: 0.08–0.63; P trend = 0.017 and OR: 0.22, 95% CI: 0.08–0.63; P trend = 0.020), respectively. Furthermore, after additional adjusting for WC, energy, first pregnancy age, number of children, history of abortion, use of anti-inflammatory drugs, and vitamin *D* supplementation in the finally adjusted model, individuals in the highest vs lowest quartiles of LLDS showed statistically significant lower risk of BrCa in all three categories (OR: 0.21; 95% CI: 0.11–0.43; P trend <0.001 for overall population, OR: 0.26; 95% CI: 0.10–0.68; P trend = 0.003 for premenopausal, and OR: 0.20; 95% CI: 0.06–0.60; P trend = 0.015 for post-menopausal women).

## 3. Discussion

The aim of our study was to investigate the relationship between the adherence to the LLDS and risk of BrCa. The results showed that adherence to a diet with a higher LLDS, which is high in vegetables, fruits, and legumes and nuts can reduce risk of BrCa in in the overall population. Also, in the highest quartile compared to the lower quartile of LLDS, a 74 and 80% reduction in the risk of BrCa was observed in premenopausal and post-menopausal women, respectively. Which, this risk reduction was greater in post-menopausal women.

Although our study examined association of LLDS and BrCa for the first time, a similar study with the same root mechanism of action was performed in relation to LLDS and risk of type 2 diabetes by Vinke et al. The result of this study indicated that LLDS can decrease the risk of type 2 diabetes in the Dutch Lifelines cohort [21]. Khani et al. published a case-control study on 278 overweight and obese women in 2020 years, which showed higher LLDS associated with better sleep quality [20].

Previous studies have also shown an association between MED and HEI dietary pattern indices, which in most respects are similar to LLDA, with a reduced risk of chronic diseases such as BrCa. Although, due to the differences that mentioned above between these indices with LLDS, the results cannot be fully generalized to our work. However, a case-control study by Turati et al. in 2018 years in Italy and Switzerland, the results showed that adherence to the Mediterranean diet significantly reduced the risk of BrCa in pre- and post-menopausal women [14]. In another study, adherence to a Western diet with a high content of meat and processed foods, sweetened foods with sugar was associated with a higher risk of BrCa (OR for the top *vs* the bottom quartile 1.46 (95% CI 1.06–2.01)), especially in premenopausal women(OR = 1.75; 95% CI 1.14–2.67) [26]. In contrast, the rich dietary pattern of olive oil, fruits and vegetables as a Mediterranean pattern (OR for the top quartile *vs* the bottom quartile 0.56 (95% CI 0.40–0.79)) was associated with a lower risk of this disease [26]. A meta-analysis consisting of cohort studies also showed a beneficial effect of the Mediterranean diet on reducing the incidence of BrCa [27]. Furthermore, Shahril et al. [13] conducted a case-control study in 2013 to examine the association of HEI with BrCa risk in 764 patients (382 BrCa cases and 382 healthy women). They concluded that, like the LLDS, a higher score of HEI reduces the risk of BrCa. The components in this study showed that individuals who eat more fruits and vegetables have a higher score and a lower risk of BrCa. Also in this study, in line with our study, the reduction in BrCa risk in postmenopausal women was significantly greater than in premenopausal women, which could be due to the effect of estrogen, which is significantly reduced at menopausal age. Studies have also shown the important role of estrogen in the risk of BrCa and changes in female fat profile [13, 28].

According to result of our study, BrCa patients significantly had lower consumption of healthy food groups including vegetables, fruits, and legumes and nuts and higher intake of coffee. In line with our study, recently in NHS Health (NHS, 1980–2012) and NHSII (1991–1991) study on 182,145 women with BrCa, the results showed that consuming more fruits and vegetables, especially cruciferous and yellow/orange vegetables (>5.5 vs. ≤2.5 servings/day HR = 0.89, 95% CI = 0.83–0.96; *p*_trend_ = 0.006), was associated with a significant reduction in BrCa [29]. In addition, recent meta-analysis studies have shown beneficial effects of fruit and vegetable intake on reducing the risk, mortality rate, and disease recurrence in these patients [30, 31].

Similarly, recently in a cohort study by Sanchez et al. with an annual follow-up of 115,802 individuals and 101 new cases of breast cancer among postmenopausal women in 2020 years, more than 1 cup of coffee per day was associated with a lower incidence of breast cancer (HR 0.44; 95% confidence interval: 0.21, 0.92) [32]. However, in a meta-analysis study with a total of 21 prospective studies for dose-response, 13 prospective studies showed no significant relationship between coffee consumption and BrCa risk in the nonlinear model [33]. However, when the analysis was limited to postmenopausal women, consuming four cups of coffee daily was associated with a 10% reduction in the risk of BrCa during menopause [33]. In addition, in line with our study, no significant association was found between moderate to low fish intake (2 portions per week or less) and the risk of breast cancer. However, high fish consumption (more than 4 portions per week) in adolescents and middle-aged people was associated with a 30% reduction in breast cancer [34]. Therefore, it seems that since the positive components of LLDS are rich in antioxidant compounds [35], such as omega 3 unsaturated fatty acids [36], and polyphenols [37], anti-inflammatory, and antioxidant pathways may be activated by increasing the consumption of these components and reducing the risk of BrCa [38–40]. On the other hand, these components often reduce visceral fat and stored body fat, by reducing estrogen, they can have beneficial effects on reducing the risk of BrCa.

Among the negative components of this score, red and processed meat and sugar-sweetened beverages intake were significantly higher in the case group than the control group as mentioned in our study. It is more commonly consumed in BrCa patients. Sugar-sweetened beverages in these diets also increase insulin secretion. Insulin increases the secretion of growth-promoting substances such as IGF-1 (insulin like growth factor-1) causes higher growth of tumor and cancer cells, thus increasing the risk of cancer [41]. The higher score of LLDS index seems to indicate that adherence to a healthier diet is associated with higher consumption of beneficial micronutrients, dietary antioxidants, fruits, vegetables, and phytochemicals (as one of the anti-inflammatory compounds). Therefore, a consequential reduction in the odds of cancer by controlling the proliferation and growth of cancer cells, as well as the balance between the body's oxidative and antioxidant system, may be evident [42]. In addition, in recent years, many studies have shown an association between intake of red and processed meat with an increased incidence of BrCa [43, 44]. Red and processed meat in the diet with a lower LLDS have higher saturated fatty acids, saturated fatty acids can increase some pro-inflammatory substances such as TNF-*α* in the body that causes inflammation and tissue damage, and the body's cells are prone to various cancers, including BrCa [45–47]. However, no direct study has been performed on BrCa patient with our subject.

The current study has several strengths. To the best of our knowledge, it is the first case-control study assessing the association between LLDS with risk of BrCa, which skilled people were used to interview and collect food frequency questionnaires. Our sample size was sufficient, and we tried to eliminate the effect of confounders as far as possible, by adjusting wide range of variables, and a validated questionnaire has been used. However, despite the novelty of this study, there are some limitations that should be noted. Despite the possible confounders considered in this study when analyzing, some confounders may not have been considered. Although we found evidence of an association between LLDS and BrCa, due to the retrospective design utilized in this study, we cannot prove causality of the observed associations; therefore, future prospective studies and RCTs need to confirm the veracity of this finding. In addition, data were collected using self-report modalities, which are known to be associated with over- or under-reporting. However, we sought to ameliorate this by using trained interviewers and robustly validated tools.

## 4. Conclusion

Adherence to a diet with a higher LLDS decreased the odds of BrCa, which this risk reduction was greater in postmenopausal women. However, due to the mentioned limitations, other prospective studies in this area have been suggested to be able to measure communication more fully.

## Figures and Tables

**Table 1:** Demographic, anthropometric, and lifestyle characteristics of participants across case and control groups as well as quartiles of LLDS.

Variables	Groups, mean (SD)		*P* value^a^	Quartiles of LLDS, mean (SD)		*P* value^a^
	Case (*n* = 253)	Control (*n* = 267)		Q1	Q4	
Age, y	48.91 (10.46)	47.13 (10.08)	0.062	48.21 (10.28)	48.86 (10.67)	0.957
BMI^b^, kg/m^2^	29.61 (4.55)	29.07 (5.39)	0.222	29.19 (4.88)	29.50 (5.10)	0.970
Waist- circumference (cm)	101.15 (96.39)	96.39 (13.25)	<0.001	98.34 (10.70)	97.89 (12.68)	0.439
Physical activity (Met.h/wk)	33.18 (6.11)	32.70 (5.20)	0.336	32.53 (4.88)	33.12 (5.89)	0.513
Smoking (yes), *n* (%)	(3.2) 8	(3.4) 9	0.894	3 (2.5)	3 (2.5)	0.690
Marriage age, y	19.43 (5.02)	18.98 (4.48)	0.296	19.28 (5.22)	19.00 (4.14)	0.939
First pregnancy age, y	22.29 (5.32)	20.35 (4.19)	<0.001	21.92 (5.04)	20.88 (4.89)	0.334
Child number	2.92 (1.43)	2.54 (1.59)	0.005	2.73 (1.46)	2.79 (1.54)	0.782
Abortion history (yes), *n* (%)	(37.2) 94	(29.2) 78	0.049	40 (33.6)	43 (35.2)	0.927
Menopausal status (post-menopausal), *n* (%)	115 (45.5)	114 (42.7)	0.527	48 (21)	59 (25.8)	0.652
Hormone replacement therapy (yes), *n* (%)	13 (5.1)	25 (10.9)	0.016	4 (9.5)	13 (31)	
Family history of breast cancer (yes), *n* (%)	(5.5) 14	(4.5) 12	0.594	6 (5.0)	9 (7.4)	0.479
Family history of cancer (yes), n (%)	(26.9) 68	(20.7) 55	0.097	27 (22.7)	29 (23.8)	0.801
Benign breast diseases history (yes), *n* (%)	(7.9) 20	(5.3) 14	0.224	9 (7.6)	6 (4.9)	0.712
Inflammatory disease history (yes), *n* (%)	(12.6) 32	(13.2) 35	0.863	12 (10.1)	17 (13.9)	0.068
Vitamin D supplement (yes), *n* (%)	(14.6) 37	(24.3) 65	0.005	23 (19.3)	21 (17.2)	0.177
Ever use of OCP (yes), *n* (%)	(49.8) 126	(56) 149	0.156	54 (45.4)	67 (54.9)	0.253
Anti-inflammatory drugs use (yes), *n* (%)	(10.3) 26	(17.7) 47	0.015	19 (16.0)	16 (13.1)	0.525
Education status, *n* (%)			0.518			0.143
Illiterate	(11.6) 29	(9) 24		19 (16.1)	6 (4.9)	
Low education	116 (46.6)	(50.4) 134		54 (45.8)	65 (53.3)	
Higher education	(42) 105	(40.6) 108		45 (38.1)	51 (41.8)	
Exposure to sunlight during the day (min)			0.215			0.398
Less than 30 minutes	(28.5) 72	(36) 96		30 (25.2)	44 (36.1)	
60–30 minutes	(32.4) 82	(25.5) 68		42 (35.3)	33 (27.0)	
120–60 minutes	(17) 43	(17.2) 46		19 (16.0)	15 (12.3)	
More than 120 minutes	(22.1) 56	(21.3) 57		28 (23.5)	30 (24.6)	

^a^Obtained from ANOVA for continuous variables and Chi-square for aategorical variables. ^b^BMI: body mass index.

**Table 2:** Dietary intakes of study participants across case and control groups as well as quartiles of LLDS.

	Groups, mean (SD)		*P* value^a^	Quartiles of LLDS, mean (SD)		*P* value^a^
	Case (*n* = 253)	Control (*n* = 267)		Q1	Q4	
Energy (kcal/d)	2753.45 (798.02)	2464.1 (607.43)	<0.001	2684.42 (770.14)	2592.07 (669.86)	0.589
Carbohydrate (g/d)	372.54(7.01)	343.54 (5.49)	0.001	357.55 (102.14)	362.94 (97.10)	0.859
Protein (g/d)	80.08 (1.49)	88.47 (1.56)	<0.001	78.33 (23.85)	92.51 (26.36)	<0.001
Fat (g/d)	108.24 (2.55)	91.82 (2.20)	<0.001	110.10 (48.52)	92.50 (28.38)	0.006
SFA (g/d)	32.92 (11.26)	29.20 (10.53)	<0.001	32.31 (13.21)	29.99 (9.44)	0.418
MUFA (g/d)	32.29 (13.29)	37.24 (15.97)	<0.001	33.25 (16.54)	24.92 (13.36)	<0.001
PUFA (g/d)	20.48 (10.35)	24.49 (13.29)	<0.001	26.21 (14.65)	18.95 (7.79)	<0.001
Cholesterol (mg/d)	293.52 (135.55)	261.88 (139.27)	0.009	275.98 (131.08)	286.87 (146.13)	0.850
Fiber (g/d)	37.96 (19.28)	39.89 (18.58)	0.247	40.19 (22.91)	38.76 (13.40)	0.417
Sodium (mg/d)	4740.74 (1811.95)	4307.06 (1898.50)	0.008	4847.81 (2163.35)	4265.17 (1454.76)	0.073
Potassium (mg/d)	3766.23 (1224.29)	4297.22 (1261.12)	<0.001	3484.51 (1195.27)	4725.62 (1184.66)	<0.001
Phosphor (mg/d)	1482.87 (492.60)	1617.48 (485.35)	0.002	1367.58 (472.82)	1745.82 (509.14)	<0.001
Iron (mg/d)	20.28 (9.96)	16.34 (6.06)	<0.001	18.38 (7.87)	18.50 (7.79)	0.917
Calcium (mg/d)	1215.79 (463.90)	1335.27 (458.76)	0.003	1084.30 (452.25)	1453.21 (498.15)	<0.001
Magnesium (mg/d)	370.06 (119.89)	402.91 (133.15)	0.003	335.84 (106.98)	442.72 (119.72)	<0.001
Zinc (mg/d)	11.76 (3.82)	12.95 (4.05)	0.001	11.49 (3.95)	13.52 (3.65)	<0.001
Vitamin C (mg/d)	159.16 (89.15)	197.87 (78.89)	<0.001	147.07 (82.51)	222.93 (84.78)	<0.001
Folate (mcg/d)	485.57 (168.28)	455.20 (163.07)	0.037	465.79 (186.66)	468.61 (130.00)	0.977
Vitamin B12 (mcg/d)	5.53 (3.87)	6.70 (4.53)	0.002	6.46 (5.76)	6.09 (2.84)	0.740
Vitamin E (mg/d)	17.64 (13.16)	23.59 (17.54)	<0.001	22.53 (21.15)	18.38 (9.66)	0.232
Vitamin D (mcg/d)	2.04 (3.44)	2.7 (3.06)	0.012	1.46 (1.87)	2.41 (3.27)	<0.001

Abbreviations; SFA, saturated fatty acid. ^a^Obtained from ANOVA.

**Table 3:** Dietary consumption of the 12 components included in the Lifelines Diet Score (LLDS) in grams per 1000 kcal among participants study (base case and control groups) and quartiles of LLDS.

	Groups, mean (SD)		*P* value^a^	Quartiles of LLDS, mean (SD)		*P* value^a^
	Case (*n* = 253)	Control (*n* = 267)		Q1	Q4	
LLDS score	34.72 (5.85)	37.42 (5.32)	**<0.001**	28.66 (2.19)	43.87 (2.78)	**<0.001**
Positive components						
Vegetables	113.60 (55.70)	128.86 (48.96)	**0.001**	89.47 (37.69)	159.81 (51.43)	**<0.001**
Fruits	168.43 (85.70)	187.44 (67.71)	**0.005**	140.70 (76.29)	225.03 (77.74)	**<0.001**
Whole grain products	36.07 (29.73)	33.32 (30.08)	0.295	24.04 (21.60)	41.82 (26.12)	**<0.001**
Legumes and nuts	12.55 (10.20)	16.63 (9.66)	**<0.001**	11.59 (10.30)	19.15 (9.85)	**<0.001**
Fish	4.50 (5.07)	5.39 (5.52)	0.058	3.25 (3.86)	7.10 (7.05)	**<0.001**
Oils and soft margarines	0.30 (0.72)	0.38 (0.73)	0.206	0.04 (0.11)	0.76 (1.05)	**<0.001**
Unsweetened dairy	194.18 (106.59)	197.69 (116.36)	0.721	147.86 (87.44)	242.81 (117.06)	**<0.001**
Coffee	0.02 (0.08)	0.00 (0.02)	**0.008**	0.00 (0.02)	0.02 (0.06)	0.211
Tea	1.23 (0.94)	1.21 (0.79)	0.845	1.16 (1.00)	1.26 (0.76)	0.784
Negative components						
Red and processed meat	12.98 (8.86)	11.01 (6.49)	**0.004**	15.91 (10.00)	9.81 (6.19)	**<0.001**
Butter, hard margarines	14.26 (8.42)	13.92 (8.76)	0.659	18.59 (9.50)	9.44 (5.73)	**<0.001**
Sugar-sweetened beverages	33.24 (37.08)	22.63 (23.57)	**<0.001**	45.55 (43.33)	16.18 (11.63)	**<0.001**

^a^Obtained from ANOVA.

**Table 4:** Odds ratio (OR) and 95% confidence interval (CI) for breast cancer based on quartiles of food-based Lifelines Diet Score (LLDS).

	Quartiles of score^*∗∗*^				P for trend
	Q1	Q2	Q3	Q4	
	≤31	32–35	36–40	≥41	
Overall					
Crude model	1.00 (ref)	0.54 (0.30–0.99)	0.45 (0.25–0.79)	0.31 (0.17–0.56)	<0.001
Model 1^*∗*^	1.00 (ref)	0.52 (0.28–0.95)	0.42 (0.23–0.74)	0.28 (0.15–0.52)	<0.001
Model 2^†^	1.00 (ref)	0.36 (0.18–0.71)	0.37 (0.19–0.70)	0.21 (0.11–0.43)	<0.001

Premenopausal					
Crude model	1.00 (ref)	0.78 (0.36–1.67)	0.43 (0.21–0.88)	0.35 (0.16–0.77)	0.003
Model 1^*∗*^	1.00 (ref)	0.93 (0.42–2.04)	0.41(0.19–0.85)	0.38 (0.17–0.85)	0.003
Model 2^†^	1.00 (ref)	0.51 (0.19–1.30)	0.33 (0.13–0.80)	0.26 (0.10–0.68)	0.003

Postmenopausal					
Crude model	1.00 (ref)	0.32 (0.11–0.87)	0.41 (0.16–1.09)	0.23 (0.08–0.63)	0.017
Model 1^*∗*^	1.00 (ref)	0.31 (0.11–0.87)	0.43 (0.16–1.16)	0.22 (0.08–0.63)	0.020
Model 2^†^	1.00 (ref)	0.30 (0.10–0.94)	0.41 (0.14–1.21)	0.20 (0.06–0.60)	0.015

^*∗∗*^Binary logistic regression was used to obtain OR and 95% CI. ^*∗*^Model 1: adjusted for age and BMI. ^†^Model 2: waist circumference, energy, first pregnancy age, number of children, history of abortion, hormone replacement therapy, use of anti-inflammatory drugs, and vitamin D supplementation.

## Data Availability

Data are available upon request due to privacy/ethical restrictions.
